# Using Voice-to-Voice Machine Translation to Overcome Language Barriers in Clinical Communication: An Exploratory Study

**DOI:** 10.1007/s11606-024-08641-w

**Published:** 2024-02-12

**Authors:** Patricia Hudelson, François Chappuis

**Affiliations:** 1 Department of Primary Care Medicine, Geneva University Hospitals, Geneva, Switzerland; 2 Faculty of Medicine, University of Geneva, Geneva, Switzerland

**Keywords:** machine translation, clinical communication, language barriers

## Abstract

**Background:**

Machine translation (MT) apps are used informally by healthcare professionals in many settings, especially where interpreters are not readily available. As MT becomes more accurate and accessible, it may be tempting to use MT more widely. Institutions and healthcare professionals need guidance on when and how these applications might be used safely and how to manage potential risks to communication.

**Objectives:**

Explore factors that may hinder or facilitate communication when using voice-to-voice MT.

**Design:**

Health professionals volunteered to use a voice-to-voice MT app in routine encounters with their patients. Both health professionals and patients provided brief feedback on the experience, and a subset of consultations were observed.

**Participants:**

Doctors, nurses, and allied health professionals working in the Primary Care Division of the Geneva University Hospitals, Switzerland.

**Main Measures:**

Achievement of consultation goals; understanding and satisfaction; willingness to use MT again; difficulties encountered; factors affecting communication when using MT.

**Key Results:**

Fourteen health professionals conducted 60 consultations in 18 languages, using one of two voice-to-voice MT apps. Fifteen consultations were observed.

Professionals achieved their consultation goals in 82.7% of consultations but were satisfied with MT communication in only 53.8%. Reasons for dissatisfaction included lack of practice with the app and difficulty understanding patients. Eighty-six percent of patients thought MT-facilitated communication was easy, and most participants were willing to use MT in the future (73% professionals, 84% patients). Experiences were more positive with European languages. Several conditions and speech practices were identified that appear to affect communication when using MT.

**Conclusion:**

While professional interpreters remain the gold standard for overcoming language barriers, voice-to-voice MT may be acceptable in some clinical situations. Healthcare institutions and professionals must be attentive to potential sources of MT errors and ensure the conditions necessary for safe and effective communication. More research in natural settings is needed to inform guidelines and training on using MT in clinical communication.

## BACKGROUND

Language barriers are common in medicine and can have a negative impact on quality of care and patient safety.^1–4^ The recommended strategy to ensure healthcare equity and patient safety for foreign language speaking patients is to use professional interpreter services.^5–9^ However, a number of factors contribute to the underuse of professional interpreters, including cost, availability, scheduling difficulties, and underestimation of patients’ language skills.^10–16^ With the increasing use of smartphones and tablets in healthcare,^17,18^ there has been a growing interest in the role that language translation apps might play in overcoming language barriers in healthcare and reducing interpreting-related costs.^19–23^

A “bewildering diversity of apps” ^24^ have been developed to overcome language barriers, both fixed-phrase translators and general machine translation (MT) apps, which may be rules-based, statistical, or deep learning-based (neural).^25^ Fixed-phrase translators propose pre-translated sentences that are then returned in the patient’s language, in either text or audio. General machine translation (MT) apps such as Google Translate or Microsoft Translate, and MT devices such as Pocketalk^26,27^ or Jarvisen^28^ offer voice-to-voice machine translation, which involves speech recognition and transcription, translation of the transcript, and speech generation of the translation.

Both types of apps have their strengths and limitations. The translation quality of fixed-phrase apps is generally reliable, provided they have been produced by professional interpreters. However, because such apps contain a finite number of mostly declarative sentences and closed questions, communication tends to be limited and phrases cannot be reformulated if the listener has trouble understanding. While fixed-phrase translators may be useful when interpreters are unavailable and for low-stakes, everyday conversations,^29^ some users have found them too time-consuming to use in relation to their expected benefits.^30^

MT apps and devices have potential to allow unlimited and more natural exchanges and tend to offer more languages than fixed-phrase apps but can require considerable effort on the part of users to initiate and carry out multi-turn conversations.^31^ In addition, numerous concerns have been raised about the accuracy of MT,^32–36^ which can vary considerably depending on the languages involved, speakers’ speech patterns, and conversation content. In one systematic review of MT in healthcare, most of the studies reviewed concluded that “MT error rates were currently unacceptable for actual deployment in health settings”.^37^

A move from statistical (SMT) to neural machine translation (NMT) models has significantly improved the quality of MT results. Whereas SMT looks for statistical patterns and uses probabilities in words and phrases to make translations, NMT examines translated phrases to identify linguistic patterns and structures which are then used to predict translation outputs on new data. NMT tends to be more accurate than SMT due to its ability to learn more diverse and complex language patterns.^38^

Most studies (both before and after introduction of NMT) have evaluated the translation accuracy of scripted text or speech.^39–42^ Very few studies have been conducted using voice-to-voice MT in natural, unscripted settings,^43,44^ conditions that pose additional challenges such as accent and dialect recognition, fast or complex speech, and ambient noise.^45,46^

Several authors have suggested that the use of certain speech patterns may reduce the risk of translation errors with voice-to-voice MT (e.g., using short, complete sentences and avoiding technical language and colloquialisms).^47–50^ However, we found no studies that specifically examined whether clinicians and patients in real-life situations are able to successfully adjust their speech behavior, and whether such adjustments allow for satisfactory communication when using MT.^51^

MT apps are already being used informally and unofficially by healthcare professionals for languages and situations where interpreters are not easily available.^52,53^ As MT becomes more accurate and accessible, it may become tempting to forego the costs and inconveniences of scheduling human interpreters and rely on MT more broadly. This underscores the need for more research on the use of MT in everyday clinical practice and guidance on when and how such apps might be used safely and efficiently.^54^ Towards this aim, we explored the use of voice-to-voice MT in routine clinical encounters to identify conditions and practices that may affect communication with MT.

## METHODS

### Study Context

The project was conducted in the Primary Care Division at the Geneva University Hospitals (HUG). The HUG is a 2000-bed, public hospital group, serving a socially, culturally, and linguistically diverse population of over 500,000.^55^ At the HUG, about half of patients are of non-Swiss nationality and speak more than 70 different languages. About 12% of patients speak no French at all.^56^ Community interpreters (in-person and over-the-phone) have been available to HUG staff since 1999, and a range of actions have been developed to facilitate timely and appropriate use of interpreter services.^57^ Use of MT apps is currently neither officially encouraged nor prohibited, but anecdotal evidence suggests widespread use when interpreters are unavailable or impractical.

The Primary Care Division consists of several units providing outpatient consultations for problems of primary care medicine^58^ and is the hospital Division with the greatest number of interpreter missions at the HUG. We chose this Division because we were interested in the opinions and experiences of health professionals and patients who are accustomed to using interpreters and could reflect on the comparative advantages and disadvantages of using a translation app to communicate.

### Study Participants

All health professionals (doctors, nurses, allied health professionals) working in the Primary Care Division were eligible to participate in the study. Participants were recruited through several methods. Unit heads were asked to propose staff who might be interested and available to participate, who were then contacted directly. The study was also presented in a weekly training session for residents, who were invited to participate in the study. Social workers and dieticians were contacted individually to explain the study and invite them to participate. In all instances, the study objectives were explained, anticipated difficulties were discussed, and the translation app and device were demonstrated.

### App Selection

Participants were requested to use either the Microsoft Translator app^59^ on their (personal or professional) android smartphone or the translation device Pocketalk W.^60^ Microsoft Translate (MST) is a free app that provides voice-to-voice translation for a wide range of languages. While several such apps exist, we chose this app for its user-friendly interface that facilitates two-way conversations, and for the option to choose among different voices for audio translations (male/female; accent). Pocketalk W (PW) is a purchasable translation device providing voice-to-voice translation for a wide range of languages and that can be used with Wi-Fi or cellular data. We proposed the Pocketalk as an alternative to Microsoft Translator for participants who were unable or preferred not to use their professional or personal cell phones for translation.

Both MST and PW are certified compliant with the USA Health Insurance Portability and Accountability Act (HIPAA) which sets standards for protection of health information, and the EU GDPR regulation which sets standards for all sensitive personal data including race, religion, political affiliations, sexual preferences, biometric or genetic data, and any other information relating to health.^61,62^ To further enhance data privacy, health professionals were instructed to decline voice clip contributions for review (in the app settings).

### Study Procedures

Participants (health professionals) were asked to conduct at least 5 consultations using the selected MT app or device, so that they had a chance to become familiar with the app. While most volunteers had used Google Translate, none were familiar with Microsoft Translator. Volunteers were provided with basic instructions on how to install and open the app, and how to select languages and tap the mic before speaking. They were advised to speak in complete sentences and to use plain language.

Health professionals were free to choose the consultations in which they would use the app or device but were asked to select languages for which both speech recognition and audio translation were available (voice-to-voice translation), and to avoid consultations where they anticipated an emotionally charged discussion or informed consent discussions. These minimal instructions were designed to mimic what might happen in real-world practice, but at the same time avoiding situations where communication is likely to be particularly difficult or high-stakes.

At the end of each consultation, participants filled a brief questionnaire that included 8 closed questions, plus space for open comments (see Box 1). In addition, HPs were requested to ask 3 closed questions to their patients (Box 2).

**Box 1** Post MT-use questionnaire for health professionals.

**Table:** 

1. Translation device used (Microsoft Translator/Pocketalk W) 2. Patient’s language 3. Did you understand the patient sufficiently (Yes/No) 4. Do you think the patient understood you sufficiently? (Yes/No) 5. Were you able to achieve your goals for the consultation? (Yes/No) 6. In your opinion, are next steps clear to the patient? (Yes/No) 7. How satisfied are you with today’s consultation? (Very dissatisfied/Somewhat dissatisfied; Somewhat satisfied; Very satisfied) 8. If an interpreter was not available, would you be willing to communicate with this patient again using the translation application? (Yes/No) 9. Other comments?	

**Box 2** Questions asked to the patient.

**Table:** 

Asked by the clinician: 1. How did you find our communication today, using the translation application? 2. (Very easy; Somewhat easy; Somewhat difficult; Very difficult) 3. To talk about intimate or private matters, which would you prefer, an interpreter or a translation app? (Interpreter/Translation app/Both are acceptable) 4. If an interpreter was unavailable, would you be willing to communicate with me again using the translation app? (Yes/No) Asked by the observer: 1. Did you sufficiently understand the professional? (Yes/No) 2. Do you think the professional understood you sufficiently? (Yes/No) 3. Are the next steps clear to you (regarding your health)? (Yes/No) 4. How satisfied were you with today’s consultation? (Very dissatisfied/Somewhat dissatisfied/Somewhat satisfied/Very satisfied) 5. If an interpreter was not available, would you be willing to communicate with this professional again using the translation app? (Yes/No) 6. To talk about intimate or private matters which would you prefer, an interpreter or the translation app? (Interpreter/Translation app/Both are acceptable) 7. Is there anything else you would like to say about your experience today?	

Consultations could be planned or unplanned, with or without prebooked interpreters.

Interpreter services used by the hospital were informed of the project, and for consultations where an interpreter had already been booked, the interpreter was asked to wait outside the consultation in case the health professional was unable to adequately communicate with the patient using the translation app. For unplanned consultations, participants were instructed to call a telephone interpreter in the case of communication difficulties.

As a complement to the questionnaire responses, PH observed a small number of planned consultations where the apps were used (and where an interpreter was pre-booked). PH explained to patients that their health professional would be using the app to communicate, and that the interpreter would be available in the case of communication difficulties. PH obtained verbal consent to observe the consultation and to ask a few brief questions after the consultation. Patients were informed that no health-related or identifying information would be collected, only information pertaining to use of the translation app.

Observations focused on whether the health professional and patient seemed comfortable using the app, whether the professional and patient made eye contact while speaking, and what, if any, strategies were used to ensure understanding (Box 3). Obvious translation errors and any difficulties encountered were also noted. After observing the consultation, PH asked patients a few brief questions, using either the app or an interpreter to translate (Box 2).

**Box 3** Observation checklist.

**Table:** 

• Does the HP explain and demonstrate the app to the patient? • Do the HP & patient maintain eye contact? • Does the HP speak in simple phrases? • Does the HP use simple language? • Does the HP verify the patient’s understanding? • Does the HP verify his/her own understanding? • Does the HP reformulate when necessary? • Does the HP use pen and paper to compliment or clarify the translation? • What technical difficulties were encountered? • What translation errors occurred? • What other difficulties were encountered?	

### Data Analysis

Data analysis included descriptive statistics of patients’ and health professionals’ answers to questionnaire items and summaries of observed speech practices and difficulties.

### Ethical Approval

While research ethics review is typically not required for quality improvement activities that are within professional practice, we submitted our project to the Geneva Cantonal of the Research Ethics Commission (CCER) who considered it exempt because the aim is outside the scope of the law.

## RESULTS

Fourteen health professionals conducted 60 consultations (4 with PW, 56 with MST) in 18 languages. Fifteen consultations were observed (2 with PW, 13 with MST). No patients refused the observer presence. Health professionals included 5 doctors, 6 nurses, and 3 allied health professionals.

All four consultations attempted with the PW device (Albanian, Tamil, Italian, English) were wholly unsatisfactory due to technical difficulties. Speech recognition tended to be poor, which led to nonsensical translations. In addition, audio translations were delayed and sometimes absent, probably due to unstable Wi-Fi or cellphone networks. Users also thought the PW device and its interface were awkward. Due to these difficulties, we decided to abandon the PW. Below, we present results from the 52 consultations using the voice-to-voice option in MST (in 4 cases, text translation was used because voice-to-voice translation was not available for the selected language).

### Questionnaire Responses

Health professionals (HPs) used MST in 52 consultations and 13 languages. Thirty-four consultations involved European languages, including English, Bulgarian, Spanish, Portuguese, Romanian, Russian, and Ukrainian. Eighteen consultations involved non-European languages, including Arabic, Bengali, Chinese, Hindi, Tamil, and Turkish.

Overall, HPs successfully achieved their goals in 43/52 consultations (82.7%) but were satisfied with communication in only 28/52 (53.8%). Spontaneous reasons given for dissatisfaction were lack of practice with the app (their own and patients’), which could lead to poor translations and slow down communication.

HPs understood patients sufficiently in 37/52 consultations (71.2%) and thought that patients understood them sufficiently in 40/52 consultations (76.9%). Two-way understanding occurred in 34/52 consultations (65.4%). HPs thought that follow-up was clear for patients in 44/52 (84.6%).

Totals vary for patients’ responses because health professionals did not always remember to ask patients to answer the questions. Thirty-six out of 41 patients (87.8%) thought MT-facilitated communication was easy, and most participants were willing to use MST again: 71.2% of professionals (37/52) and 88.0% of patients (37/42). Seventy-seven percent (23/30) of patients thought the app would be preferable or equal to an interpreter for discussing intimate or sensitive topics with their health professional.

Experiences were more negative for non-European languages (Table 1), mainly due to non-recognition and poor translation of patients’ speech.

**Table 1:** Overview of Questionnaire Results

Questionnaire responses	All languages (*n* = 52)	European languages (*n* = 34)	Non-European languages (*n* = 18)
Health professionals (HPs)			
Achieved consultation goals	43/52 (82.7%)	32/34 (94.1%)	11/18 (61.1%)
Understood patient sufficiently	37/52 (71.2%)	30/34 (88.2%)	7/18 (38.9%)
Thought patient understood sufficiently	40/52 (76.9%)	30/34 (88.2%)	10/18 (55.6%)
Bidirectional understanding	34/52 (65.4%)	28/34 (82.4%)	6/18 (33.3%)
Satisfied with communication	28/52 (53.8%)	24/34 (70.6%)	4/18 (22.2%)
Willing to use MT again	37/52 (71.2%)	29/34 (85.3%)	8/18 (44.4%)
Thought follow-up was clear for patient	44/52 (84.6%)	32/34 (94.1%)	13/18 (72.2%)
Patients			
MT communication was easy	36/41 (87.8%)	27/27 (100%)	9/14 (64.3%)
Satisfied with communication	10/11 (90.9%)	9/9 (100%)	9/18 (50.0%)
Willing to use MT again	37/42 (88.1%)	28/28 (100%)	9/14 (64.3%)
MT is equal or better than interpreters for intimate subjects	23/30 (76.7%)	18/22 (81.8%)	5/8 (62.5%)

#### Open-Ended Comments on the Questionnaire

Thirty-six HPs wrote brief comments on the questionnaire form. Sixteen noted that their patients’ speech was poorly translated (Arabic, Turkish, Tamoul, non-native speaker of Russian); 8 commented on circumstances where the app worked well (with practice it gets easier; works well for simple exams, when using simple phrases, with patients who speak standardized language); 6 noted that their patient had difficulty learning to use the app; 2 said they found it difficult to use the app for emotional discussions; and 4 commented that communication went well despite the occasional translation error.

### Observations

PH observed 15 consultations (Arabic, English, Portuguese, Romanian, Spanish, Turkish, Ukrainian, Russian).

In two consultations (a Romanian-speaking Roma patient and a Turkish-speaking patient, both illiterate), an interpreter was called to ensure successful communication. In both cases, patients were reluctant to try using the app, were visibly flustered and upset, and had difficulty remembering to tap the mic and to speak in short turns.

When using MST, speakers tended to look at the listener just before tapping the mic, then looked at the phone to verify that their speech was correctly recognized. Speakers often watched the listener while the text and audio translations were produced, which allowed them to monitor the listener’s reaction and detect any comprehension problems.

Speech recognition problems and translation errors occurred when speech was disfluent (fillers, stutters, pauses), when speakers used only intonation to indicate a question that was then translated as an affirmation (e.g., “You don’t have hypertension?”), with some numbers (e.g., “one, two, three” translated as 123), when using non-standard dialects (e.g., Maghrebi Arabic) or mixing words from different languages (e.g., a Spanish speaker who used the French work “rendez-vous” instead of “cita” for an appointment). Speakers sometimes forgot to tap the mic or spoke before the mic was activated which also contributed to poor or incomplete speech recognition.

Speech recognition and translation errors were quickly noticed and communicated through facial expressions (furrowed brow, laughter). When this occurred, both health professionals and patients generally either reformulated or asked for clarification. Occasionally, listeners would ignore a poor translation if overall understanding was good.

The smoothest exchanges occurred when health professionals took the time to explain and demonstrate the app to patients, created an unrushed atmosphere, spoke in short turns, used simple language and visual or written supports to ensure understanding (e.g., writing down medication names or numbers). When speakers were stressed from lack of practice with the app or rushed due to time pressures, speech was more disfluent, which could lead to recognition and translation problems.

Technical issues were rare but a few times the app had trouble detecting speech, possibly due to internet connection problems. Waiting or closing and reopening the app usually corrected the problem but caused stress and interrupted the flow of communication.

#### Potential Advantages of Using a Translation App: Remarks from Participants

Several patients commented on their experience with MST after the consultation. One patient who is hard of hearing said she appreciated being able to read the translations and commented that it was the first time she had understood everything without having to ask health professionals or interpreters to repeat themselves. Another patient thought the app would help her sister be more autonomous and less dependent on her overly controlling husband for translation. A patient with prostate problems said he would be more at ease using the app to talk with his doctor about his symptoms. Several patients asked for help in downloading the app onto their phones so they could use it in other contexts.

Health professionals commented that MST would be most appropriate in consultations involving the exchange of factual information (acute problems, medicine checks, follow-up appointments, simple exams), consultations with literate patients (who could verify and correct speech recognition), in situations where there was only a partial language barrier (when one or the other spoke and understood some of the other’s language, but not enough to forego an interpreter), and potentially with patients who were known to frequently miss appointments (to avoid unnecessary billing for interpreter services). Several nurses found MST to be a welcome and superior alternative to telephone interpreters, who were not always quickly available and were often in noisy environments. Both patients and health professionals commented that the app had potential to facilitate patients’ communication autonomy and to ensure confidentiality.

#### Potential Disadvantages of Using a Translation App: Remarks from Participants

Both patients and health professionals commented that their lack of familiarity and practice with the app made communication more difficult. A few health professionals commented that communication could take even longer than with an interpreter if they had to take time out of the consultation to explain the app to patients. They also commented that having to pay attention to how they spoke (rather than relying on interpreters to make sense of their or their patients’ sometimes disordered or incomplete phrases) could be tedious at first, but that with practice it became easier.

Finally, some health professionals thought that developing a relationship and eliciting patients’ (sometimes emotional) social and illness narratives could be difficult and time-consuming because of the need to speak in relatively short (unnatural) turns.

## DISCUSSION

Participants in our study were able to communicate in a majority of interactions using voice-to-voice MT, and most patients and healthcare professionals were moderately to very satisfied with the MST-translated interactions and willing to repeat the experience in the future. However, experiences and satisfaction varied depending on the language being translated, the type of interaction, and speakers’ ability to adapt speech patterns to accommodate the app.

To our knowledge, ours is the first study to explore the use of voice-to-voice MT in real-world clinical situations, for a wide range of languages, and with health professionals and patients who are accustomed to using interpreters to communicate. We identified only two previous studies that explored the use of voice-to-voice MT in natural settings. While reactions to MT were positive, both studies were limited to a single language (Spanish) and conducted in contexts with limited or no access to interpreters, conditions that may increase the likelihood of satisfaction. ^63,64^ Health professionals and patients in our study found that voice-to-voice MT was useful and acceptable, but only for some languages and in some clinical situations.

While more experience and feedback from a wider range of medical specialties and clinical situations is needed to inform the development of guidelines for safe and effective use of MT, our preliminary results suggest that voice-to-voice MT is likely to be more successful:

- With speakers of European languages, or speakers of non-European languages who can produce and understand “standardized” forms of their language.^65^
- With speakers who are comfortable with smartphone technology
- With speakers who are able to modulate their speech to accommodate MT, in particular to speak in full sentences using plain language

Health professionals who use voice-to-voice MT need to be aware of common sources of speech recognition problems and translation errors and know how to avoid or manage them. Compared to human interpreters, voice-to-voice MT has several disadvantages, including difficulty detecting contextual clues and translating non-standard language, cultural expressions and disfluency (fillers, stutters, pauses). This underscores the importance of general communication skills for detecting and addressing potential communication problems, such as using plain language, pacing one’s speech, being attentive to nonverbal cues, verifying understanding, and using visual and written supports.

### Study Limitations

Our study has several limitations. First, we did not systematically examine the accuracy of translations produced by MST. We were not interested in specific translation errors, but rather in whether and how health professionals successfully managed communication when using MT. Although we observed that listeners signaled when strange or unclear translations were produced and that speakers responded by repeating, reformulating, or using visual aids, it is possible that undetected and potentially important misunderstandings occurred. It would be useful to examine more closely how different kinds of translation errors affect communication and understanding.

Second, we had limited feedback from patients. HPs often failed to ask feedback questions to patients, and therefore responses may not adequately reflect patient experiences. Most HPs said they simply forgot or did not have time to ask the questions, but it is possible that they chose (consciously or unconsciously) not to ask the questions in situations where communication was more difficult, and where patients may have had a more negative experience. Some patients may not have felt comfortable giving negative feedback to (or about an interaction with) their HP.

Finally, our findings are limited to a self-selected group of HPs working in a single, hospital-based primary care service, and therefore may not be relevant to other HPs or clinical contexts. More research is needed on whether and how HPs in other medical specialties and healthcare contexts can communicate effectively with patients using MT before more general guidelines and recommendations can be proposed. Nonetheless, our results suggest that under certain conditions voice-to-voice MT can be an acceptable and effective means to overcome language barriers.

## CONCLUSION

Effective communication is essential for the delivery of quality healthcare, and trained, professional interpreters continue to be the gold standard for overcoming language barriers in healthcare. Nonetheless, time and cost pressures, limited access to interpreters, and easy access to mobile translation apps have led to increased interest in and use of MT apps to overcome language barriers with patients. While voice-to-voice MT may be a potentially useful and cost-saving strategy for addressing language barriers in some clinical situations, its effective use requires an understanding of its limitations as well as significant speech adaptions. Healthcare institutions and professionals must be attentive to the potential sources of translation and communication errors and ensure the conditions necessary for effective communication.
